# Integrated epidemiological and molecular analysis of *Cryptosporidium* spp. and *Giardia duodenalis* isolates in dairy calves from Terceira Island, Azores

**DOI:** 10.1007/s00436-025-08613-x

**Published:** 2025-12-05

**Authors:** Mariana Louro, José C. T. Linhares, Carlos Augusto Pinto, Román Pino-Vera, Telmo Nunes, Jacinto Gomes, Isabel Pereira da Fonseca

**Affiliations:** 1 CIISA – Centre for Interdisciplinary Research in Animal Health, Faculty of Veterinary Medicine, University of Lisbon, Avenida da Universidade Técnica, Lisbon, 1300-477 Portugal; 2 Associate Laboratory for Animal and Veterinary Sciences (AL4AnimalS), Lisbon, Portugal; 3 Laboratório de Parasitologia, Instituto Nacional de Investigação Agrária e Veterinária (INIAV), Oeiras, 2780-157 Portugal; 4 Institute of Agricultural and Environmental Research and Technology (IITAA), University of the Azores, Angra do Heroísmo, 9500-321 Portugal; 5 UNICOL - Cooperativa Agrícola, C.R.L, Angra do Heroísmo, Portugal; 6 CBA – Biotechnology Centre of Azores, University of the Azores, Rua Capitão João d’Ávila, Angra do Heroísmo, 9700-042 Portugal; 7 Instituto Universitario de Enfermedades Tropicales y Salud Pública de Canarias, Universidad de La Laguna (ULL), Av. Astrofísico F. Sánchez, sn, La Laguna, Canary Islands 38203 Spain; 8 Departamento de Obstetricia y Ginecología, Pediatría, Medicina Preventiva y Salud Pública, Toxicología, Medicina Legal y Forense y Parasitología, Facultad de Farmacia, Universidad de La Laguna (ULL), La Laguna, Canary Islands Spain; 9 Programa de Doctorado de Ciencias Médicas y Farmacéuticas, Desarrollo y Calidad de Vida, Universidad de La Laguna (ULL), La Laguna, Spain; 10 Elvas School of Biosciences, Polytechnic Institute of Portalegre, Portalegre, Portugal; 11 VALORIZA – Research Centre for Endogenous Resources Valorization, Polytechnic Institute of Portalegre, Portalegre, Portugal

**Keywords:** Dairy calves, Prevalence, Molecular epidemiology, Zoonoses, Risk factors, Portugal.

## Abstract

*Cryptosporidium* spp. and *Giardia duodenalis* are major enteric pathogens in neonatal calves, contributing to gastrointestinal disease and raising concerns due to their zoonotic potential. Despite their global distribution, little is known about their occurrence and genetic diversity in insular regions such as the Azores archipelago (Portugal). This study investigated the prevalence, distribution, genetic diversity, and farm-level risk factors associated with these parasites in dairy calves from Terceira Island. Fecal samples (*n* = 142) from 28 farms were examined by microscopy, direct immunofluorescence assay (DFA), and PCR targeting multiple loci (*SSU rRNA*, *gp60*, *LSU rRNA* for *Cryptosporidium* spp.; *gdh*, *bg*, *tpi* for *G. duodenalis*). Sequencing enabled molecular identification of species, genotypes, and assemblages. Farm management data were collected by standardized questionnaires, and spatial analysis used farm location. Overall, 65.5% (93/142) of calves were infected with at least one parasite, with *Cryptosporidium* spp. and *Giardia* spp. detected in 42.3% (60/142) and 44.4% (63/142) of samples, respectively. Mixed infections occurred in 21.1% (30/142) of calves. Molecular characterization identified *C. parvum* (subtype IIaA15G2R1), *C. bovis*, and *C. ryanae*, as well as *G. duodenalis* assemblage E (predominant) and assemblage A (rare). Spatial mapping showed *Cryptosporidium* spp. to be widespread, while *Giardia* spp. displayed localized clusters. Use of paromomycin and higher hygiene scores were associated with reduced *Giardia* risk, whereas no risk factors were found for *Cryptosporidium*. These findings provide novel insights into protozoan infections in calves in the Azores and highlight the value of integrating molecular, spatial, and management data for targeted control strategies.

**Supplementary Information:**

The online version contains supplementary material available at 10.1007/s00436-025-08613-x.

## Introduction

*Cryptosporidium* spp. and *Giardia duodenalis* are widespread intestinal protozoan parasites that infect a broad range of vertebrate hosts, including humans and livestock. These pathogens are primarily transmitted via the fecal-oral route, either through direct contact with infected individuals or by ingestion of contaminated food or water containing infectious stages (oocysts or cysts) (Xiao 2010; Feng and Xiao 2011). In dairy calves, both parasites are recognized as major causes of neonatal diarrhea, a condition that significantly contributes to morbidity, mortality, and economic losses in cattle production systems, while also raising concerns due to their zoonotic potential (Santin 2020).

Among *Cryptosporidium* species, *C. parvum* is the main agent associated with clinical disease in calves, often resulting in watery diarrhea during the first weeks of life (Thomson et al. 2017). Other species such as *C. bovis*,* C. ryanae* and *C. andersoni* are also frequently reported in cattle but are typically associated with subclinical infections (Buchanan et al. 2025). In contrast, *G. duodenalis* is a common intestinal protozoan that infects calves of various ages, often without clinical signs (Santin 2020). Genotypically, *G. duodenalis* is divided into several assemblages, with assemblages A and B considered zoonotic and assemblage E mostly restricted to livestock (Feng and Xiao 2011).

Although numerous studies have reported the prevalence and molecular diversity of *Cryptosporidium* spp. and *G. duodenalis* in cattle worldwide (Taghipour et al. 2022; Buchanan et al. 2025), data from the Azores archipelago remain extremely limited. To date, no peer-reviewed studies have documented the molecular characterization or spatial distribution of these parasites in this region. A previous study conducted on Terceira Island reported a 31.2% (78/250) prevalence of *Cryptosporidium* spp. in calves under 60 days of age, based on modified Ziehl-Neelsen stain (Barros 2015). The infection was significantly associated with younger age groups and the presence of diarrhea. However, no molecular characterization or spatial analysis of infection patterns was performed (Barros 2015). Data regarding *G. duodenalis* in livestock from the Azores are entirely lacking.

Terceira Island plays a pivotal role in Portugal’s dairy sector. In 2024, Portugal produced approximately 1.97 billion liters of milk, of which the Azores contributed with 608.5 million liters, accounting for 30.9% of national production, according to Portugal’s National Statistics Institute (Portal do INE). Terceira Island has the second-highest milk production, following São Miguel Island in the archipelago. The region’s temperate oceanic climate, with mild year-round temperatures and consistently high humidity, combined with pasture-based farming systems, supports highly productive and sustainable dairy operations (Massot 2015; Almeida et al. 2020). Despite this agricultural prominence and the zoonotic potential of enteric protozoa, little is known about the occurrence, molecular identity, and spatial patterns of *Cryptosporidium* spp. and *G. duodenalis* infections in local livestock systems.

Therefore, this study aimed to address these gaps through an integrated investigation of *Cryptosporidium* spp. and *G. duodenalis* in dairy calves from Terceira Island, Azores. Specifically, we aimed to: (i) estimate infection prevalence at both calf and farm levels; (ii) compare diagnostic performance of microscopy, direct immunofluorescence antibody test, and PCR-based methods; (iii) identify *Cryptosporidium* species and *Giardia* assemblages via sequencing; (iv) assess their geographic distribution across parishes; and (v) evaluate associations with farm-level management practices.

## Materials and methods

### Farms, animals and sampling

Between August 2023 and May 2024, a total of 142 fecal samples were collected from dairy calves, aged between 3 days to 10 weeks old. The calves were from 28 dairy farms located in 25 parishes on Terceira Island, Azores. At each farm, a veterinarian collected five to six individual rectal samples into sterile containers. The samples were preserved by diluting them 1:1 in 5% (wt/vol) potassium dichromate and were then stored in a refrigerator at 4 °C until analysis. The number of calves sampled per farm was determined considering an expected within-farm prevalence above 30%, based on non-published field observations from local veterinarians and previous surveillance experience in the region. This sample size ensured a probability greater than 85% of detecting at least one infected animal per farm, while remaining compatible with logistical feasibility and calf availability on the sampling date. Sampling was conducted with prior informed consent from the animal owners and in accordance with ethical approval (see Ethical statement).

Individual data recorded at the time of sampling included the calf’s sex, date of birth, health status, and the presence or absence of diarrhea. Additionally, a structured questionnaire was completed by the veterinarian during each farm visit to collect farm-level information. This included general characteristics such as production system and total herd size, as well as specific management and hygiene practices, including the source of drinking water, proximity to natural watercourses, access to non-treated water, bedding material, type of calf housing (individual or group), colostrum administration, type of milk provided, vaccination status, use of paromomycin or other drugs for prevention, previous history of *Cryptosporidium* and *Giardia* infections, frequency of neonatal diarrhea, and the types of treatments typically applied.

At the end of the visit, a general hygiene score was also assigned to each farm by the veterinarian, using a scale from 0 (poor) to 10 (excellent). This information enabled the contextualization of parasite infection patterns in relation to farm-level environmental, management, and preventive practices.

### Microscopic analysis

All samples were initially processed using a concentration technique outlined by Louro et al. (2025a). The Modified Ziehl-Neelsen stain (MZN) was employed to identify *Cryptosporidium* spp. oocysts (Casemore et al. 1984), while a direct immunofluorescence antibody (DFA) test (MeriFluor^®^ *Cryptosporidium/Giardia*, Meridian Bioscience) was used for the detection of both *Cryptosporidium* oocysts and *Giardia* cysts. Each sample was analyzed under a light microscope for MZN slides and a UV light microscope for DFA slides.

### Molecular analysis

#### DNA extraction

Genomic DNA was extracted from 250 µl of each fecal sample concentrate utilizing the NZY Tissue gDNA Isolation Kit (NZYTech, Portugal), according to the specific protocol designed for stool samples. To enhance the digestion of oocysts and cysts, an overnight incubation at 60 °C was conducted with the addition of proteinase K. The extracted DNA samples were subsequently stored at −20 °C for future analyses.

#### *Cryptosporidium* spp. and *G. duodenalis* PCR amplification

All samples underwent nested PCR targeting the small subunit ribosomal RNA (*SSU rRNA*) gene to detect the presence of *Cryptosporidium*, following methodologies established by Xiao et al. (1999, 2000). Positive samples were further analyzed using restriction fragment length polymorphism (PCR-RFLP) with *SspI* and *MboII* enzymes, as described by Feng et al. (2007). The resulting digestion products were then separated on 2% agarose gels stained with GreenSafe Premium (NZYTech, Portugal) for visualization.

Samples confirmed as *C. parvum* positive were subjected to further subtyping through the sequencing of the highly polymorphic 60 kDa glycoprotein (*gp60*) gene, in accordance with the protocols described by Alves et al. (2003). Additionally, samples exhibiting unique PCR-RFLP patterns distinct from *C. parvum*, as well as *C. parvum* samples that were negative for subtype analysis, were sequenced to accurately identify or confirm the *Cryptosporidium* species present.

For a more comprehensive analysis, 12 positive samples underwent separate nested PCR amplification targeting the V8 region of the large-subunit ribosomal rRNA gene (*LSU rRNA*), as detailed by Koehler et al. (2017), followed by sequencing to further confirm the *Cryptosporidium* species involved.

For the analysis of *G. duodenalis*, all samples underwent nested PCR targeting three specific genes: glutamate dehydrogenase (*gdh*) as described by Read et al. (2004), beta-giardin (*bg*) following Lalle et al. (2005), and triose phosphate isomerase (*tpi*) as described by Sulaiman et al. (2003). Sequencing was employed for assemblage identification in one *gdh* gene-positive sample collected from each *G. duodenalis* positive farm.

Each PCR reaction was designed to incorporate 3 µL of template DNA for the initial amplification and 1 µL of the primary PCR product for the subsequent reaction. To ensure the reliability of the results, each PCR run included a positive control sample, either *C. parvum* or *G. duodenalis*, as well as a purified water aliquot serving as a negative control. The amplification products were then visualized through electrophoresis on 1.5% agarose gels stained with GreenSafe Premium (NZYTech, Portugal). Details of PCR conditions can be found in the Supplementary Tables 1 and 2.

#### Sequencing and phylogenetic analysis

PCR products were purified utilizing NZYGelpure (NZYTech, Portugal). Sequencing was performed on both strands using internal primer sets by Stabvida (Portugal). The consensus sequences were edited with MEGA12 software (Kumar et al. 2024) and compared against sequences available in GenBank. *Cryptosporidium parvum gp60* isolates were classified into subtypes following the nomenclature system established by Sulaiman et al. (2005). Phylogenetic trees for the *SSU rRNA* and *LSU rRNA* genes of *Cryptosporidium*, as well as the *gdh* gene of *G. duodenalis*, were constructed using the sequences generated in this study, supplemented with reference sequences from GenBank. The *gdh* sequences of *G. duodenalis* were aligned without gaps. In contrast, due to the non-coding nature of *Cryptosporidium SSU* and *LSU* regions and the variability in sequence lengths, both sequence groups were aligned using the MAFFT software with the L-INS-I algorithm (Katoh et al. 2005, 2019). Maximum likelihood phylogenetic trees were generated via the IQ-TREE web server (http://iqtree.cibiv.univie.ac.at) (Nguyen et al. 2015; Trifinopoulos et al. 2016). The ModelFinder feature of IQ-TREE (Kalyaanamoorthy et al. 2017) was employed to identify the optimal phylogenetic model for all trees, based on the Bayesian information criterion. To assess node support statistics, ultrafast bootstrapping (Hoang et al. 2018) was performed with 1000 replicates. The resulting phylogenetic trees were visualized and further refined using MEGA12 (Kumar et al. 2024) and R software (v4.3.1; R Core Team 2023).

### Data analysis

The data analysis was performed using R software (v4.3.1; R Core Team 2023), with a significance level of *p* ≤ 0.05 applied to all statistical tests. Descriptive statistics were used to summarize calf clinical status and farm-level characteristics. Calf age was calculated in days as the difference between sampling and birth dates. In addition, the relationship between calf age and *Cryptosporidium* species was examined.

A broad set of clinical and farm-level variables was tested for possible associations with parasite infection. Associations between infection and categorical variables (e.g., diarrhea, overall health status, or use of preventive measures) were evaluated using contingency tables, with chi-square or Fisher’s exact tests applied as appropriate. Odds ratios (ORs) and 95% confidence intervals (CIs) were calculated from 2 × 2 contingency tables using the *epitools package* (Aragon 2020). For continuous or ordinal variables (e.g., hygiene classification score), Mann-Whitney U tests (Wilcoxon rank-sum test) were used. Differences in calf age among *Cryptosporidium* species were assessed using the Kruskal–Wallis test, followed by Dunn’s post-hoc comparisons with Bonferroni correction. Although multiple variables were examined, only those showing statistically significant or borderline-significant associations with infection outcomes are presented in the results.

Geographic maps were produced using the *ggplot2* and *sf* packages (Wickham 2016; Pebesma 2018; Pebesma and Bivand 2023) to explore the spatial distribution of parasitic infections across parishes of Terceira Island. These maps included infection prevalence and the distribution of identified *Cryptosporidium* species and *Giardia* assemblages, and were used as descriptive tools to support interpretation of epidemiological patterns.

## Results

### Prevalence and diagnostic performance for *Cryptosporidium* spp. and *Giardia* spp

A total of 65.5% (*n* = 93) of calves and 100% (28/28) of the sampled farms were infected with at least one of the two protozoan parasites (*Cryptosporidium* spp. and/or *Giardia* spp.) (Table 1). *Cryptosporidium* spp. were detected in 42.3% (*n* = 60) of calves across 24 farms. Among the diagnostic methods used, PCR targeting the *SSU rRNA* gene proved to be the most sensitive for *Cryptosporidium* (40.8%; 58/142), followed by DFA (12.7%; 18/142) and modified Ziehl-Neelsen staining (9.9%; 14/142).

**Table 1:** Prevalence of *Cryptosporidium* spp. And *Giardia* spp. In calves And farms

Category	Calves (*n* = 142)		Farms (*n* = 28)	
	Nº positive (%)	95% CI	Nº positive (%)	95% CI
*Cryptosporidium* spp.				
MZN	14 (9.9%)	6.0–15.9	9 (32.1%)	17.9–50.7
DFA	18 (12.7%)	8.2–19.1	10 (35.7%)	20.7–54.2
PCR *SSU rRNA*	58 (40.8%)	33.1–49.1	24 (85.7%)	68.5–94.3
Total	60 (42.3%)	34.4–50.5	24 (85.7%)	68.5–94.3
*Giardia* spp.				
DFA	59 (41.5%)	33.8–49.8	25 (89.3%)	72.8–96.3
PCR *gdh*	23 (16.2%)	11.0–23.1	15 (53.6%)	35.8–70.5
PCR *bg*	20 (14.1%)	9.3–20.8	12 (42.9%)	26.5–60.9
PCR tpi	10 (7.0%)	3.9–12.5	9 (32.1%)	17.9–50.7
Total	63 (44.4%)	36.5–52.6	26 (92.9%)	77.4–98.0
*Cryptosporidium* spp. and/or *Giardia* spp.				
Total	93 (65.5%)	57.4–72.8	28 (100%)	87.9–100

*DFA* direct immunofluorescence assay, *MZN* modified Ziehl-Neelsen, *SSU rRNA* small subunit ribosomal RNA, *gdh* glutamate dehydrogenase, *bg* β-giardin, *tpi* triosephosphate isomerase

*Giardia* spp. was slightly more prevalent, detected in 44.4% (*n* = 63) of calves and in 92.9% (26/28) of farms. Among the four diagnostic techniques used for *Giardia* spp., DFA showed the highest sensitivity (41.5%, 59/142). Regarding molecular detection, PCR targeting the *gdh* gene (16.2%; 23/142) was more effective than PCR targeting *bg* (14.1%; 20/142) or *tpi* (7.0%; 10/142).

Mixed infections with both parasites were common, with 21.1% (*n* = 30) of calves testing positive for both *Cryptosporidium* spp. and *Giardia* spp.

### Spatial distribution of parasitic infections in calves from Terceira Island

**Fig. 1:**
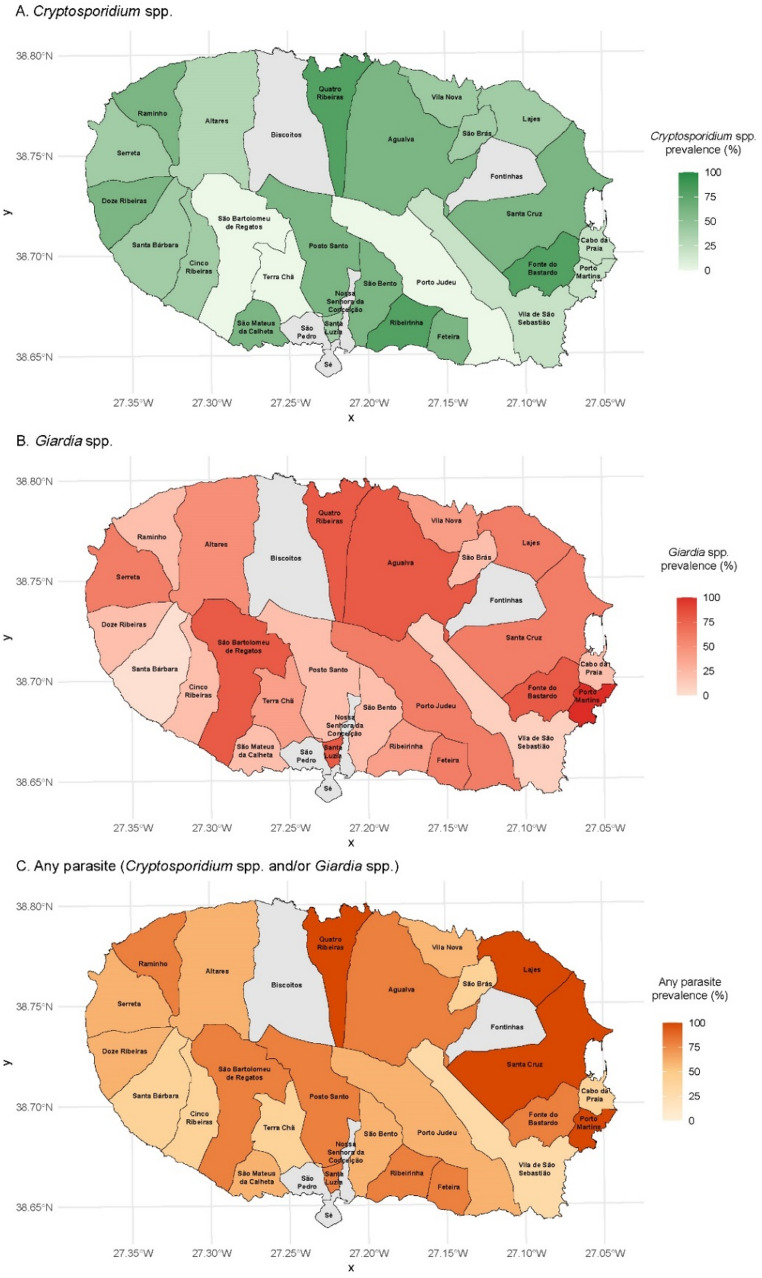
Prevalence maps of *Cryptosporidium* spp. (**A**), *Giardia* spp. (**B**), and infection with either parasite (**C**) in dairy calves from 28 farms across Terceira Island, Azores. Color gradients indicate the proportion of infected calves per farm (0–100%)

Parasitic infections were geographically widespread across Terceira Island, with infected calves detected in all surveyed parishes (Fig. 1; Supplementary Table 3). The overall prevalence of infection with at least one parasite ranged from 20% to 100% across farms, with higher values observed in Lajes, Porto Martins, Santa Cruz, Quatro Ribeiras, and one farm in Altares, where all sampled calves tested positive.

*Cryptosporidium* spp. exhibited a relatively uniform distribution, affecting calves in most regions of the island. Moderate to high prevalence (≥ 60%) was recorded in 11 farms with the highest values (80%) observed in Fonte do Bastardo, Quatro Ribeiras and Ribeirinha. In contrast, no positive cases were detected in 4 farms from São Bartolomeu de Regatos, Porto Judeu, Terra Chã, and one of the farms in Vila de São Sebastião.

*Giardia* spp. exhibited greater spatial heterogeneity compared to *Cryptosporidium* spp., with marked variation in prevalence across farms. High infection rates (≥ 80%) were recorded in seven farms, with the maximum value of 100% prevalence observed in Porto Martins. In contrast, no *Giardia-*positive calves were detected in two farms located in Santa Bárbara and Vila de São Sebastião, and low prevalence levels (20%) were observed in ten additional farms scattered across multiple parishes.

Mixed infections were particularly frequent (*n* ≥ 3) in calves from farms in Quatro Ribeiras, Agualva, and Fonte do Bastardo, reinforcing their classification as high-risk areas.

Given that only five to six calves were sampled per farm, the precision of prevalence estimates at the farm or parish level may be limited.

### Farm management practices

Farm-level data were collected from all 28 participating farms using a standardized questionnaire (Table 2). Most farms operated under semi-intensive systems (60.7%; *n* = 17), and the majority were of medium size (100–250 animals; 46.4%; *n* = 13). Water quality appeared to be well managed: 92.9% (*n* = 26) of farms used controlled water sources, and 78.6% (*n* = 22) reported no access to non-controlled water sources. However, 64.3% (*n* = 18) of farms were located near natural watercourses, which could serve as potential sources of environmental contamination.

**Table 2:** Description of farm management and hygiene variables obtained through questionnaire (*n* = 28)

Variables	*n*	%
Production system		
Intensive	11	39.3%
Semi-intensive	17	60.7%
Farm size (total animals)		
Small (< 100)	8	28.6%
Medium (100–250)	13	46.4%
Large (> 250)	7	25.0%
Nearby natural water sources		
Yes	18	64.3%
No	10	35.7%
Controlled water source		
Yes	26	92.9%
No	2	7.1%
Access to non-clean water source		
Yes	6	21.4%
No	22	78.6%
Bedding		
Cement	2	7.1%
Wood	1	3.6%
Straw	4	14.3%
Soil	21	75.0%
Previous occurrence of *Cryptosporidium*		
Yes	24	85.7%
No	4	14.3%
Previous occurrence of *Giardia*		
Yes	0	0%
No	28	100.0%
Frequency of diarrheas (first week of life)		
Occasionally	7	25.0%
Sometimes	12	42.9%
Frequently	4	14.3%
Almost always	5	17.9%
Use of Paromomycin for prevention		
Yes	18	64.3%
No	10	35.7%
Vaccination for other diseases		
Yes	12	42.9%
No	16	57.1%

Soil was the most commonly used bedding material (75%; *n* = 21), and maternal colostrum and whole milk were the primary feed sources during the neonatal period in all farms. A substantial proportion of farmers (85.7%; *n* = 24) reported previous presence of *Cryptosporidium* spp. on their farms, but none had any prior reports of *Giardia* spp. infection.

Concerning neonatal calf health, frequent or almost constant diarrhea during the first week of life was reported by 32.2% (*n* = 9) of farmers, with an additional 42.9% (*n* = 12) reporting it sometimes. Paromomycin was used as a preventive measure in 64.3% (*n* = 18) of farms, although only 42.9% (*n* = 12) reported vaccinating against other neonatal pathogens, namely bovine viral diarrhea (BVD) and infectious bovine rhinotracheitis (IBR). None of the surveyed farms reported using vaccines specifically targeting other neonatal diarrhea pathogens such as rotavirus, coronavirus, or enterotoxigenic *Escherichia coli*. A smaller number of farms also used additional prophylactic treatments, such as toltrazuril.

### Associations between farm management and parasitic infection

Statistical associations between parasite infection and selected farm-level variables are summarized in Table 3. A significantly lower prevalence of *Giardia* infection was observed in calves from farms that used paromomycin as a preventive measure (OR = 0.43; 95% CI: 0.21–0.86; *p* = 0.021). A similar association was found for infection with any parasite (*Cryptosporidium* spp. and/or *Giardia* spp.), with paromomycin use associated with reduced odds of infection (OR = 0.35; 95% CI: 0.15–0.76; *p* = 0.009). No statistically significant association was found between paromomycin use and *Cryptosporidium* spp. infection alone (*p* = 0.213).

**Table 3:** Associations between parasitic infection in calves and selected farm management variables

Variable	Parasite	Comparison	Odds Ratio (OR)	95% CI	*p*-value
Prevention with Paromomycin	*Giardia*	Yes vs. No	0.43	[0.21–0.86]	0.021
	*Cryptosporidium*	Yes vs. No	0.62	[0.30–1.24]	0.213
	Both parasites	Yes vs. No	0.35	[0.15–0.76]	0.009
Hygiene score	*Giardia*	↑ score ↓ infection risk	—	—	0.011
	*Cryptosporidium*	No association	—	—	0.902
	Any parasite	↑ score ↓ infection risk	—	—	0.073

Farm hygiene score was also associated with *Giardia* infection: calves from farms with higher hygiene scores were significantly less likely to be infected (*p* = 0.011). A borderline association was observed for infection with any parasite (*p* = 0.073), while no association was found for *Cryptosporidium* infection (*p* = 0.902).

### Molecular and phylogenetic analysis

#### *Cryptosporidium* spp

Molecular analysis revealed the presence of three *Cryptosporidium* species: *C. parvum* (*n* = 32), *C. bovis* (*n* = 17) and *C. ryanae* (*n* = 9). Sequencing of the *gp60* gene showed the presence of only one *C. parvum* genotype: IIa A15G2R1 (*n* = 24).

Eight *C. parvum* samples were negative by PCR of the *gp60* gene. However, the species was confirmed by sequencing of the *SSU rRNA* gene. Also, two DFA positive samples, remained negative during PCR analysis.

*SSU rRNA* sequence analysis showed that all *C. bovis* sequences were 100% identical to GenBank accession number OQ456121. Seven *C. parvum* sequences were identical to GenBank accession number AB513875. One sequence was 100% identical to *C. parvum* (OQ361939), however, it was distinct from the others for having a shorter sequence length. For *C. ryanae*, seven sequences were also identical to GenBank accession number OQ456123, and one identical to KY711520, differing by this last one having an extra nucleotide.

For more in-depth analysis, 12 samples were selected for the PCR of the *LSU rRNA* gene: an unidentified *Cryptosporidium* species (from low sequence quality), one *C. bovis*, two *C. parvum* and eight *C. ryanae* samples. Four samples had no amplification: both *C. parvum* and two *C. ryanae*. The *C. bovis* sequence was 99.74% identical to GenBank accession number KY882320. The unidentified *Cryptosporidium* sequence was 100% identical to those from previously identified *C. ryanae* samples. These sequences were 100% identical to GenBank accession number OL865382 (*Cryptosporidium* sp.).

**Fig. 2:**
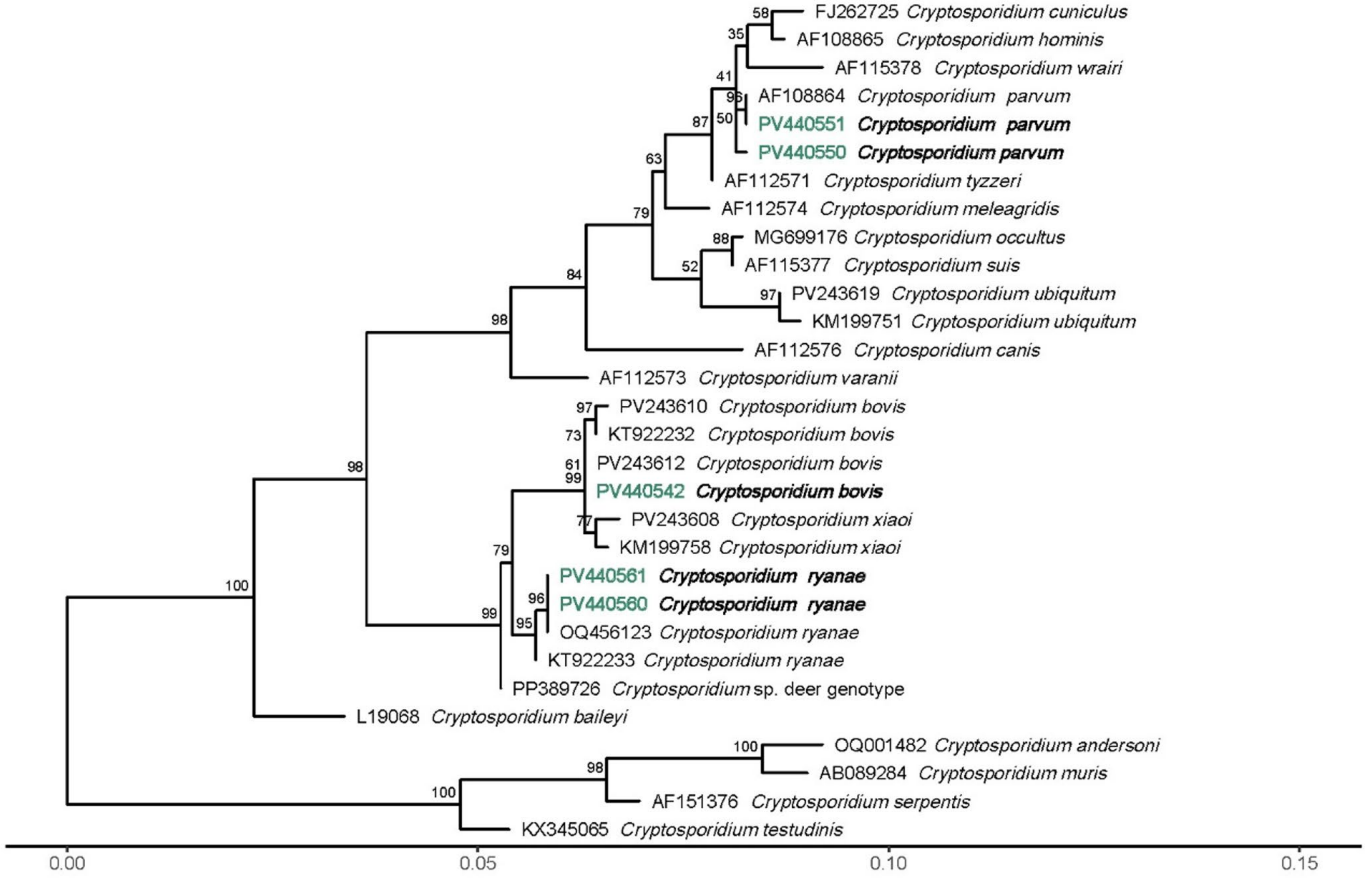
Phylogenetic tree of *Cryptosporidium* spp. *SSU rRNA* gene

**Fig. 3:**
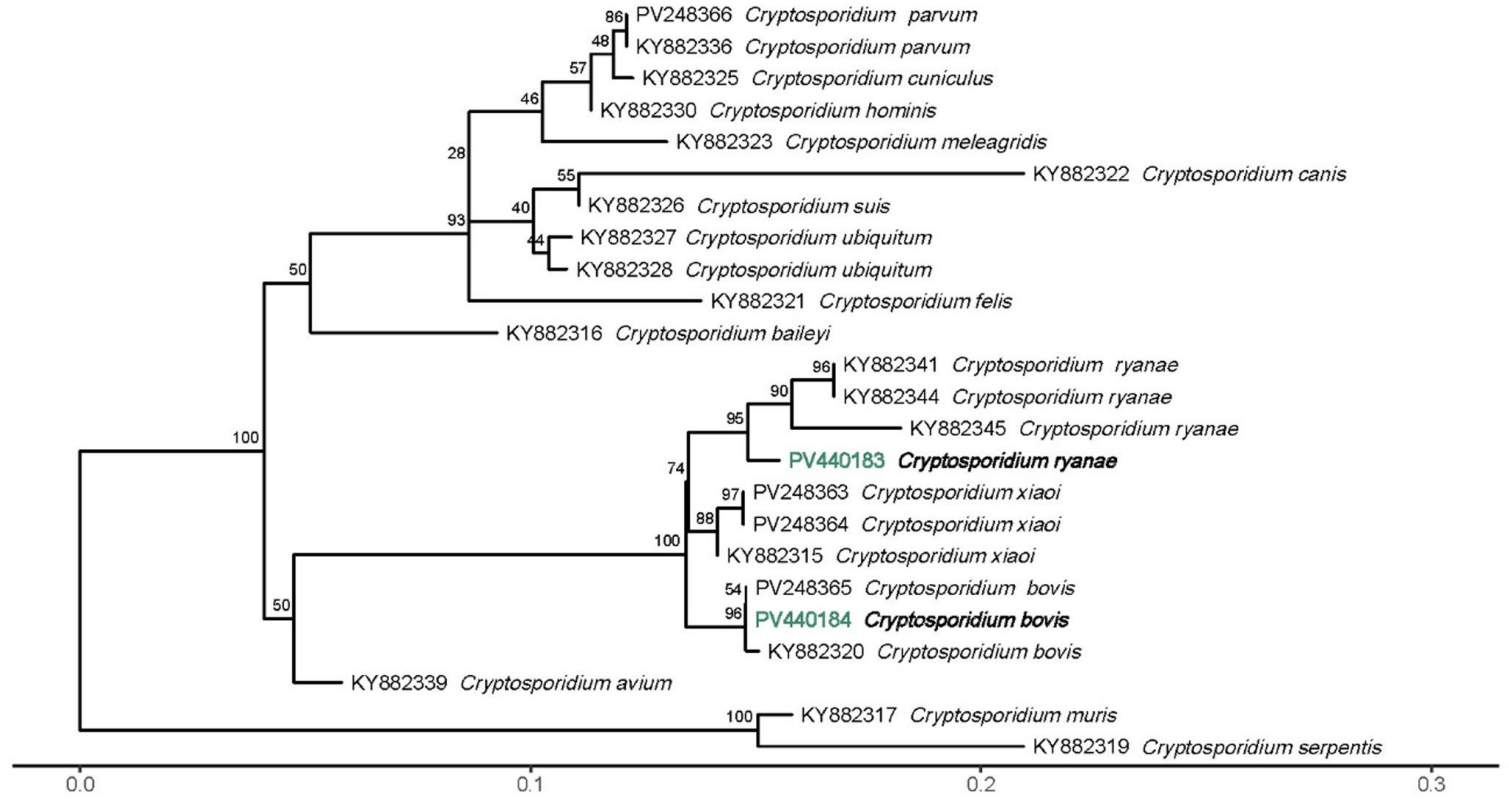
Phylogenetic tree of *Cryptosporidium* spp. *LSU rRNA* gene

One representative sequence from each distinct group was chosen to calculate a phylogenetic tree for both *SSU rRNA* and *LSU rRNA* genes. While for the *SSU* tree (Fig. 2), the sequences clustered with representative sequences for each *Cryptosporidium* species, in the *LSU* tree (Fig. 3), the *C. ryanae* sequence from this study creates a distinct cluster.

All *Cryptosporidium* sequences obtained were submitted to GenBank under accession numbers PV440542 - PV440574 (*SSU rRNA*), PV440177 - PV440184 (*LSU rRNA*) and PV446750 - PV446773 (*gp60*).

#### Giardia duodenalis

Overall, PCR of the *gdh* gene was more sensitive, with only 4 samples being negative for this gene and positive for *bg* and/or *tpi* genes. There was no amplification in any of the three genes tested in 11 DFA *Giardia*-positive farms. Since the remaining 15 farms had all at least one sample positive for the *gdh* gene, this marker was used for *G. duodenalis* assemblage identification in the farms. Among PCR-positive samples, sequence analysis revealed the presence of two distinct assemblages: A (*n* = 1) and E (*n* = 14). Diversity of 4 distinct groups was observed within the assemblage E sequences: one group with 7 sequences, another with 5, and two groups with one sequence each.

**Fig. 4:**
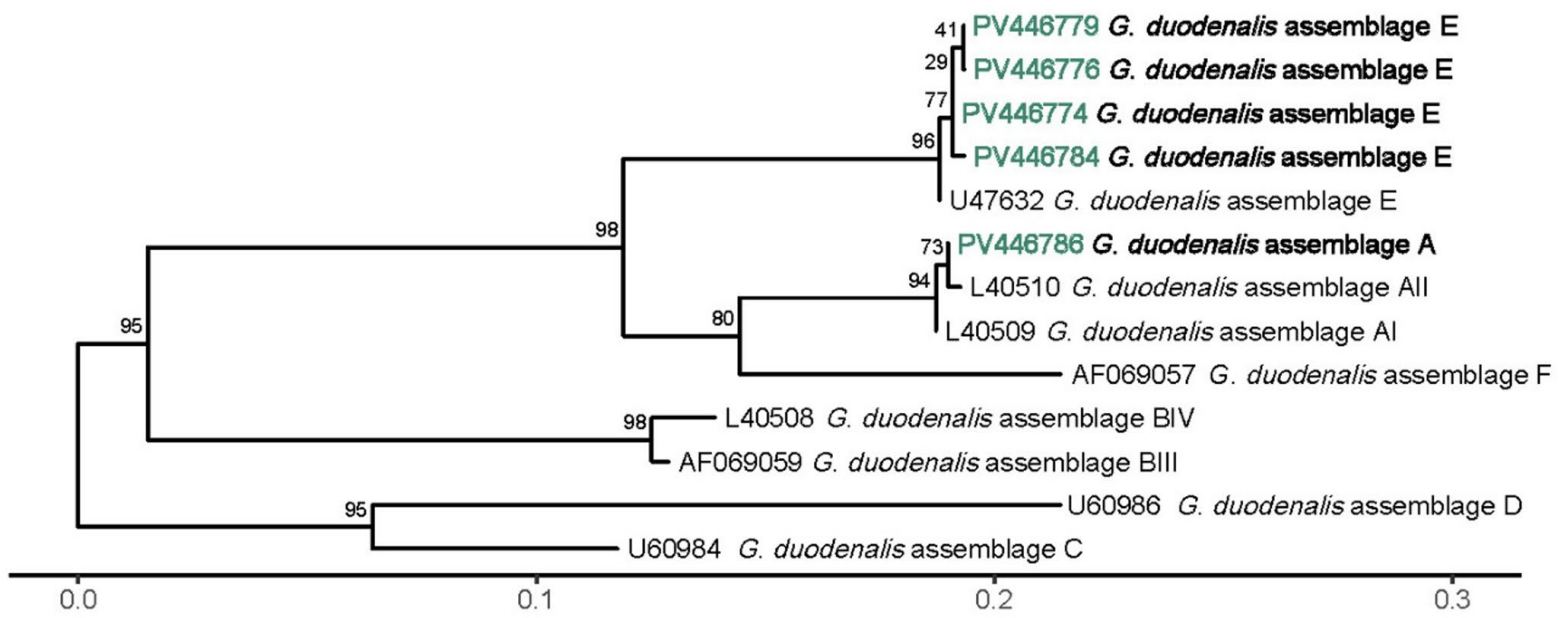
Phylogenetic tree of *G. duodenalis gdh* gene

One representative sequence from each distinct group and assemblage was chosen to calculate a phylogenetic tree. *Giardia duodenalis* assemblage A and E sequences clustered with their respective group (Fig. 4).

*Giardia duodenalis gdh* gene sequences obtained were submitted to GenBank under accession numbers PV446774 - PV446788.

### Age distribution of *Cryptosporidium* species

Analysis of calf age by *Cryptosporidium* species revealed an age-related pattern (Table 4). Calves infected with *C. parvum* were significantly younger (mean: 19.1 days), while *C. bovis* and *C. ryanae* were more frequently detected in older calves, with median ages of 31.2 and 39.1 days, respectively. The age range also widened progressively across these species, with *C. ryanae* detected in calves up to 74 days old. The age differences between *C. parvum*, *C. bovis* and *C. ryanae* were statistically significant (Kruskal-Wallis, X^2^ = 9.16, df = 2, *p* = 0.010). Post-hoc analysis showed that calves infected with *C. parvum* were significantly younger than those infected with *C. ryanae* (*p* = 0.027), while differences between *C. parvum* and *C. bovis* (*p* = 0.128) and between *C. bovis* and *C. ryanae* (*p* = 0.606) were not significant after Bonferroni correction.

**Table 4:** Age distribution (in days) of calves infected with different *Cryptosporidium* species

*Cryptosporidium* species	*n*	Mean age (days)	SD	Min (days)	Max (days)
*C. parvum*	32	19.1	7.5	6	40
*C. bovis*	17	31.2	11.8	15	54
*C. ryanae*	9	39.1	16.5	21	74

### Geographic distribution of *Cryptosporidium* species and *Giardia duodenalis* assemblages

**Fig. 5:**
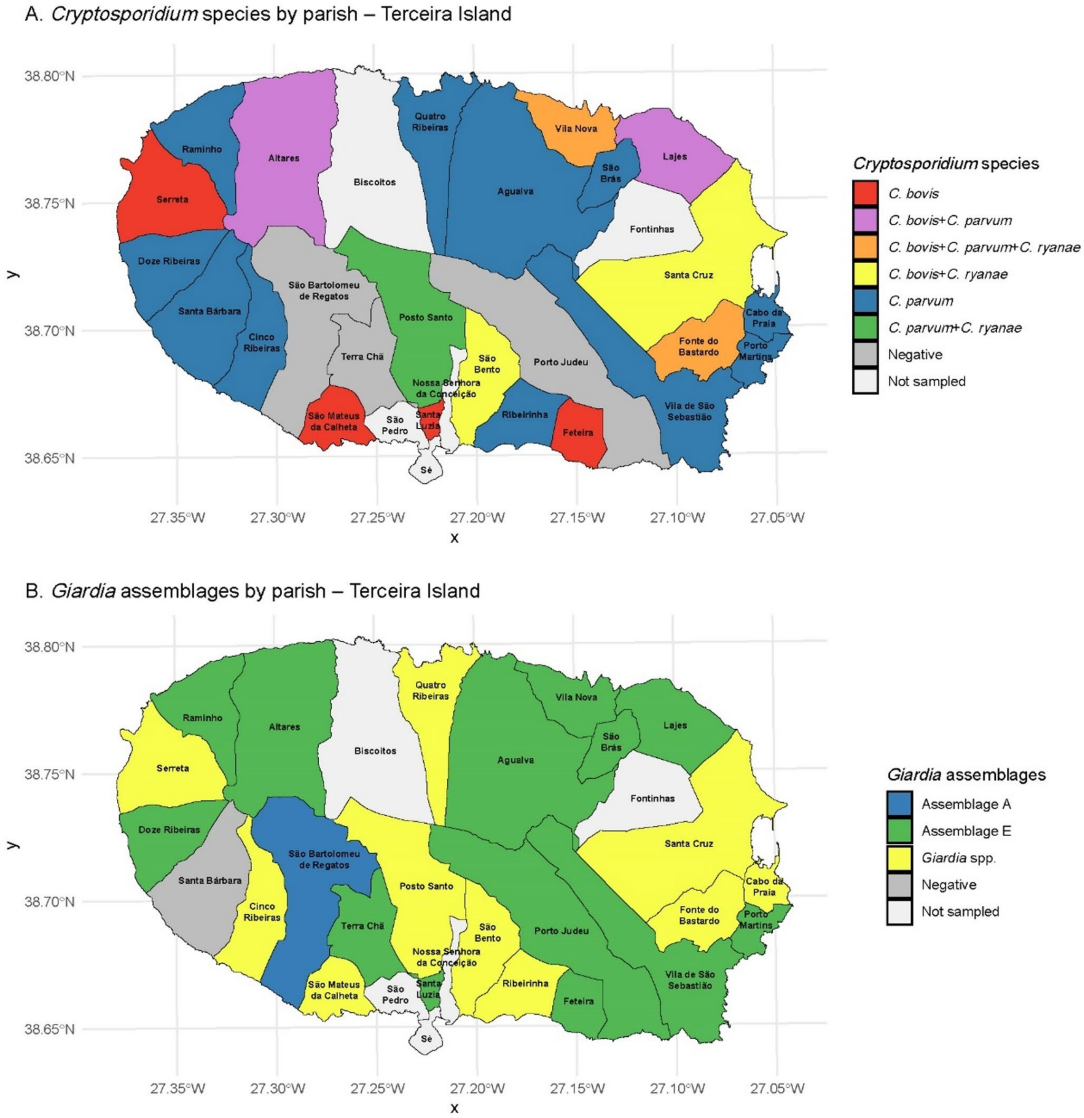
Maps of the distribution of *Cryptosporidium* species (**A**) and *Giardia* assemblages (**B**) in dairy calves from Terceira Island, Azores

To better understand the spatial distribution of parasite diversity, the occurrence of *Cryptosporidium* species and *G. duodenalis* assemblages was mapped by parish across Terceira Island (Fig. 5).

For *Cryptosporidium*, the most frequently detected species was *C. parvum*, identified in 16 parishes, either alone or in co-infection with *C. bovis* and/or *C. ryanae*. Farms with the highest species diversity, where all three species were detected, were located in Fonte do Bastardo and Vila Nova. In contrast, some farms presented only *C. parvum*, while others showed exclusive detection of *C. bovis*.

The spatial distribution of *G. duodenalis* assemblages showed that assemblage E was the most common, detected in the majority of positive parishes. Assemblage A was identified in a single farm in São Bartolomeu de Regatos. Several farms had *Giardia*-positive samples for which assemblage identification was not achieved.

These spatial results demonstrate considerable variation in the composition of *Cryptosporidium* species and *G. duodenalis* assemblages across farms on Terceira Island.

## Discussion

This study provides the first integrated analysis of the prevalence, molecular diversity, and spatial distribution of *Cryptosporidium* spp. and *G. duodenalis* in dairy calves from Terceira Island, Azores. The high overall prevalence of infection (65.5%) and the detection of both parasites in nearly all farms highlight the widespread circulation of these protozoa in the region.

The prevalence of *Cryptosporidium* spp. (42.3%) and *Giardia* spp. (44.4%) observed in this study was higher than the global average prevalence in pre-weaned calves, estimated at 37.5% for *Cryptosporidium* spp. (Buchanan et al. 2025) and 34% for *G. duodenalis* (Taghipour et al. 2022). These elevated values suggest a substantial circulation of both parasites in the studied population. However, similar prevalence rates have been reported in other European settings. For instance, Castro-Hermida et al. (2006) observed up to 58.5% prevalence of *Cryptosporidium* in calves under one month of age and 56.7% for *Giardia* in animals aged one to five months in Galicia, Spain. Likewise, a study in Poland found *Cryptosporidium* spp. in 45.3% of calves up to 16 weeks of age, while in Norway, *G. duodenalis* was detected in 49% of dairy calves (Hamnes et al. 2006; Rzeżutka and Kaupke 2023). These findings indicate that the values observed in Terceira Island, although high, fall within the upper range of what has been reported across Europe.

Environmental and management factors likely contribute to the high infection rates observed. In Azores, the mild oceanic climate, characterized by high humidity and frequent rainfall, favors the survival and dissemination of cysts and oocyst in the environment (Fox et al. 2015; Marbella et al. 2022). Furthermore, dairy farming on Terceira Island is predominantly pasture-based and small-scale, often involving shared grazing areas and limited infrastructure, which can increase environmental contamination and facilitate transmission (Silva et al. 2019; Almeida et al. 2020; Medeiros et al. 2021). Such conditions, combined with close contact among young animals, create a favorable setting to early and repeated exposure to enteric protozoa.

In this study, clinical signs, particularly diarrhea, were frequently reported in neonatal calves, with more than 75% of farms indicating at least occasional cases during the first week of life. However, no consistent association was found between diarrhea at the time of sampling and infection with either *Cryptosporidium* spp. or *G. duodenalis*. Previous studies have yielded mixed results regarding these associations. Some have reported a higher prevalence of *Cryptosporidium* spp. in diarrheic calves (*p* < 0.01) and a significant correlation between *G. duodenalis* infection and diarrhea in pre-weaned calves (OR = 2.61; 95% CI: 1.50–4.55) (Taghipour et al. 2022; Gao et al. 2023). In contrast, other studies, including one conducted in Sweden, found no significant differences in the prevalence of either *Cryptosporidium* spp. or *G. duodenalis* between diarrheic and healthy calves, suggesting a more ubiquitous distribution of these parasites (Björkman et al. 2003). The lack of association observed here may reflect the cross-sectional nature of the study, which did not always include animals at the peak of clinical signs, neither the possible presence of other pathogens, such as rotavirus or *Escherichia coli*, which were not screened in this work. Nonetheless, infections by these protozoa can impair calf growth and productivity, with severe neonatal cryptosporidiosis reducing live-weight gain in a Scottish herd, and studies in the United States reporting decreased weight gain in calves infected with either parasites (Urie et al. 2018; Shaw et al. 2020).

Mixed infections were also frequent in our study, with 21.1% of calves positive for both *Cryptosporidium* spp. and *G. duodenalis*, underscoring the common early-life exposure of calves to multiple enteric pathogens. In contrast, a Norwegian study reported a much lower coinfection rate of just 9.1% among dairy calves (Hamnes et al. 2006). Moreover, a recent review on coinfections in young ruminants (Delling and Daugschies 2022) highlights that simultaneous infections involving *Cryptosporidium*, *Giardia*, rotavirus, *Escherichia coli*, and other enteropathogens are often observed in diarrheic calves, and may be underreported due to limited diagnostic scope. Together, these findings support the concern that mixed infections may exacerbate disease severity, leading to more persistent diarrhea, dehydration, and reduced growth performance in affected animals (Santin 2020).

The comparative evaluation of diagnostic methods confirmed that molecular tools, particularly PCR targeting the *SSU rRNA* gene, were the most sensitive approach for detecting *Cryptosporidium* spp., outperforming traditional microscopy techniques. For *G. duodenalis*, the DFA yielded the highest detection rates, corroborating previous findings that highlight the superior performance of immunologic methods over molecular techniques, especially in field samples with variable cyst loads (Taghipour et al. 2022). In contrast, PCR targeting the *gdh*, *bg* and *tpi* genes demonstrated lower sensitivity, which may be attributed to suboptimal primer binding, low parasite DNA concentrations, or the presence of PCR inhibitors commonly found in fecal samples (Coklin et al. 2011). These technical limitations, widely reported in parasitological diagnostics, can lead to false negatives and underestimation of true prevalence. Because molecular characterization relied only on PCR-positive samples, some DFA-positive but PCR-negative cases were missed, meaning that the diversity of *G. duodenalis* assemblages detected in this study may be underestimated. Consequently, the predominance of assemblage E observed here should be interpreted as applying only to the genotyped subset and may have been influenced by locus or primer performance. The application of multiple, complementary diagnostic methods in this study was therefore critical to improving detection accuracy and providing a more comprehensive picture of protozoan infection dynamics in the surveyed population.

Spatial analysis revealed different distribution patterns between *Cryptosporidium* spp. and *G. duodenalis*. *Cryptosporidium* spp. were more uniformly distributed across Terceira Island, with consistent detection in most parishes and relatively stable prevalence levels between farms. This pattern is consistent with the high environmental resistance of *Cryptosporidium* oocysts, which can survive for prolonged periods under diverse climatic conditions and spread efficiently through contaminated bedding, shared pastures, and water sources (Olson et al. 1999; Hamilton et al. 2018). In contrast, *Giardia* spp. exhibited a more heterogeneous and clustered distribution, with high-prevalence farms concentrated in specific areas, likely reflecting the greater susceptibility of *Giardia* cysts to environmental factors (Olson et al. 1999; Hamilton et al. 2018). These spatial differences align with the management associations observed: *Giardia* spp. prevalence was significantly lower in farms using paromomycin and those with higher hygiene scores, while no similar effect was seen for *Cryptosporidium* spp. However, this association should be interpreted with caution, as paromomycin use was reported as a preventive practice rather than an evidence-based control measure, and its administration raises concerns regarding antimicrobial stewardship. The observed correlation likely reflects broader management differences between farms rather than a direct protective effect of the drug. The lack of significant risk factors for *Cryptosporidium* spp. infection probably reflects its environmental resilience and widespread dissemination, leading to uniform exposure among farms. Oocysts can persist for long periods under the humid island conditions, while relatively similar husbandry practices across Terceira may have further limited between-farm variability and the ability to detect management-related differences. The detection of high-prevalence clusters for both parasites in specific parishes suggests localized hotspots, possibly linked to environmental contamination or regional management practices.

Additionally, the complete absence of previous reports of *Giardia* spp. infection on these farms is noteworthy, especially given the high prevalence detected in this study. This may reflect underdiagnosis due to historically limited surveillance or the use of low-sensitivity diagnostic methods. The findings highlight the need to raise awareness among farmers and veterinarians regarding *Giardia* spp. as a relevant enteric pathogen in calves, and emphasize the importance of including it in future diagnostic and control programs.

Molecular analysis identified *C. parvum* as the dominant species, with all isolates belonging to the zoonotic subtype IIaA15G2R1. This finding is consistent with another study in calves from Portugal and global data reporting *C. parvum* as the most prevalent species in pre-weaned calves and identifying IIaA15G2R1 as the dominant subtype in Europe and worldwide (Louro et al. 2024; Bellinzona et al. 2024; Buchanan et al. 2025). Notably, this genotype is also frequently implicated in human cryptosporidiosis outbreaks, particularly those linked to contact with livestock or contaminated water (Chalmers et al. 2019). Its detection in the present study reinforces the potential public health relevance of calf infections in Terceira, Azores, particularly in rural communities with close contact between animals and people.

In addition to *C. parvum*, the species *C. bovis* and *C. ryanae* were detected at lower frequencies, reflecting a broader spectrum of *Cryptosporidium* circulating in the calf population. These species were primarily identified in older animals, in line with previously described age-related shifts in species occurrence (Santin 2020; Díaz et al. 2021; Jang et al. 2023). This temporal pattern likely reflects the typical infection dynamics in calves, where *C. parvum* predominates during the neonatal period and is gradually replaced by less pathogenic species as animals mature (Díaz et al. 2021). While the clinical significance of *C. bovis* and *C. ryanae* remains uncertain, their detection may indicate continued environmental exposure and low-level transmission within herds. To our knowledge, this is the first report of *C. ryanae* in calves in Portugal, having previously only been identified in red deer and lambs in the country (Figueiredo et al. 2023; Louro et al. 2025b).

The spatial distribution of *Cryptosporidium* species revealed farms with high diversity, where all three species co-occurred. Additional patterns of co-infection were also observed, however, the majority of farms presented only *C. parvum* or *C. bovis*, suggesting variable exposure patterns and possibly different sources of contamination. Interestingly, similar results were reported in lambs from northern Portugal, where greater *Cryptosporidium* species diversity was also observed, although *C. parvum* remained the most prevalent, followed by *C. bovis* (Louro et al. 2025b). Notably, *C. bovis* was only detected in lambs that cohabited with cattle, suggesting potential cross-species transmission (Louro et al. 2025b). Although no specific data were collected in this study regarding co-grazing or housing with other animal species, such interactions are common in mixed or small-scale farming systems. It is therefore plausible that similar interspecies transmission dynamics may occur in calves from farms where other ruminants are present. These parallels between calves and lambs may reflect shared environmental or management-related risk factors across livestock systems in Portugal and further support the need for broader, cross-species surveillance studies to better understand transmission pathways and epidemiological links.

Molecular analysis of *G. duodenalis* revealed an overwhelming predominance of assemblage E, consistent with its host specificity for ruminants and its global distribution in calves (Taghipour et al. 2022). This assemblage was widely detected across Terceira Island and showed intra-assemblage diversity, with four distinct sequence groups identified through *gdh* gene analysis, suggesting local genetic variation within or between farms. Only one isolate belonged to assemblage A, a zoonotic genotype, indicating zoonotic risk in the studied population (Heyworth 2016). Still, its presence, although rare, highlights the potential for human exposure and underscores the importance of continued surveillance. A limitation of the study was the inability to sequence additional *Giardia*-positive samples, which may have led to underestimation of genotypic diversity and zoonotic potential.

It should also be acknowledged that the limited number of calves sampled per farm may have affected the precision of prevalence estimates and the strength of detected associations. Nevertheless, the consistent spatial and epidemiological patterns observed still provide valuable base line information, particularly in the context of an insular production system where such data are scarce, and can be further refined through future, larger-scale studies.

Taken together, the results of this study highlight the complex epidemiology of *Cryptosporidium* spp. and *G. duodenalis* in dairy calves in Terceira, shaped by interactions between parasite biology, environmental factors, and farm-level practices. The detection of zoonotic genotypes, the presence of co-infections, and the identification of high-prevalence clusters reinforce the importance of a One Health approach that considers animal, environmental, and human health as interconnected. Furthermore, the combined use of multiple diagnostic tools, molecular typing, and spatial mapping proved essential to expand our knowledge of parasite diversity and transmission dynamics in this region. These findings are particularly relevant for insular and pasture-based systems, where infrastructure limitations may hinder effective disease control. Continued surveillance efforts by environmental sampling, and farmer education, will be crucial for improving parasite management, reducing animal health impacts, and mitigating potential zoonotic risks.

## Conclusion

This study provides the first comprehensive assessment of *Cryptosporidium* spp. and *G. duodenalis* infections in dairy calves in Terceira Island, integrating epidemiological, molecular, and spatial data. The high prevalence of infection, widespread detection across farms, and identification of both zoonotic (*C. parvum*, assemblage A) and livestock-adapted genotypes (*C. bovis*, *C. ryanae*, assemblage E) underscore the complexity of protozoan parasite transmission in calf-rearing systems.

Importantly, the study identified associations between improved hygiene practices and paromomycin use with reduced *Giardia* spp. prevalence. This observation, however, should be interpreted as an association rather than a causal relationship. While it reflects current farm practices in the region, the preventive use of antibiotics is not advocated and must be considered in light of antimicrobial resistance concerns. Overall, these findings highlight the importance of good hygiene management as a sustainable and responsible strategy for parasite control and support the implementation of targeted measures to minimize environmental contamination and calf exposure, particularly during the vulnerable neonatal period.

By addressing a critical knowledge gap in an underrepresented geographic context, this work contributes valuable baseline data for regional disease surveillance and veterinary decision-making. Further research in other Azores islands, including longitudinal designs, environmental sampling, and multi-pathogen approaches, is recommended to better understand infection dynamics, monitor control effectiveness over time, and evaluate the broader One Health implications of zoonotic transmission.

## Supplementary Information

Below is the link to the electronic supplementary material.

Supplementary Material 1 (DOCX 46.6 KB)

## Data Availability

The data supporting this study are available within the article and its Supplementary Information. Sequence data have been deposited in GenBank under accession numbers PV440542–PV440574, PV440177–PV440184, PV446750–PV446773, and PV446774–PV446788.

## References

[CR1] Almeida AM, Alvarenga P, Fangueiro D (2020) The dairy sector in the Azores islands: possibilities and main constraints towards increased added value. Trop Anim Health Prod 53:40. 10.1007/s11250-020-02442-z 33231751 10.1007/s11250-020-02442-z PMC7685184

[CR2] Alves M, Xiao L, Sulaiman I et al (2003) Subgenotype analysis of *Cryptosporidium* isolates from humans, cattle, and zoo ruminants in Portugal. J Clin Microbiol 41:2744–2747. 10.1128/JCM.41.6.2744-2747.2003 12791920 10.1128/JCM.41.6.2744-2747.2003 PMC156540

[CR3] Aragon TJ (2020) epitools: Epidemiology Tools. R package version 0.5–10.1 https://CRAN.R-project.org/package=epitools

[CR4] Bellinzona G, Nardi T, Castelli M et al (2024) Comparative genomics of Cryptosporidium parvum reveals the emergence of an outbreak-associated population in Europe and its spread to the United States. Genome Res 34:877–887. 10.1101/gr.278830.123 38977307 10.1101/gr.278830.123 PMC11293552

[CR5] Björkman C, Svensson C, Christensson B, de Verdier K (2003) *Cryptosporidium parvum* and *Giardia intestinalis* in calf diarrhoea in Sweden. Acta Vet Scand 44:145. 10.1186/1751-0147-44-145 15074627 10.1186/1751-0147-44-145 PMC1831560

[CR6] Buchanan R, Wieckowski P, Matechou E et al (2025) Global prevalence of *Cryptosporidium* infections in cattle: a meta-analysis. Curr Res Parasitol Vector-Borne Dis 7:100264. 10.1016/j.crpvbd.2025.100264 40487329 10.1016/j.crpvbd.2025.100264 PMC12144461

[CR7] Casemore DP, Armstrong M, Jackson B et al (1984) Screening for *Cryptosporidium* in stools. Lancet 323:734–735. 10.1016/S0140-6736(84)92245-1

[CR8] Castro-Hermida JA, Carro-Corral C, González-Warleta M, Mezo M (2006) Prevalence and intensity of infection of *Cryptosporidium* spp. and *Giardia duodenalis* in dairy cattle in Galicia (NW Spain). Journal of Veterinary Medicine, Series B 53:244–246. 10.1111/j.1439-0450.2006.00946.x 16732884 10.1111/j.1439-0450.2006.00946.x

[CR9] Chalmers RM, Robinson G, Elwin K, Elson R (2019) Analysis of the *Cryptosporidium* spp. and *gp60* subtypes linked to human outbreaks of cryptosporidiosis in England and Wales, 2009 to 2017. Parasites & Vectors 12:95. 10.1186/s13071-019-3354-6 30867023 10.1186/s13071-019-3354-6 PMC6417012

[CR10] Coklin T, Farber JM, Parrington LJ et al (2011) Immunomagnetic separation significantly improves the sensitivity of polymerase chain reaction in detecting *Giardia duodenalis* and *Cryptosporidium* spp. in dairy cattle. J Vet Diagn Invest 23:260–267. 10.1177/104063871102300210 21398445 10.1177/104063871102300210

[CR11] de Barros SVA (2015) Contribuição para o estudo da criptosporidiose em vitelos de explorações leiteiras da ilha Terceira, Açores. Dissertation, Universidade de Lisboa, Faculdade de Medicina Veterinária

[CR12] Delling C, Daugschies A (2022) Literature review: coinfection in young ruminant livestock—*Cryptosporidium* spp. and its companions. Pathogens 11:103. 10.3390/pathogens11010103 35056051 10.3390/pathogens11010103 PMC8777864

[CR13] Díaz P, Navarro E, Remesar S et al (2021) The age-related *Cryptosporidium* species distribution in asymptomatic cattle from North-Western Spain. Animals 11:256. 10.3390/ani11020256 33498538 10.3390/ani11020256 PMC7909547

[CR14] Feng Y, Ortega Y, He G et al (2007) Wide geographic distribution of *Cryptosporidium bovis* and the deer-like genotype in bovines. Vet Parasitol 144:1–9. 10.1016/j.vetpar.2006.10.001 17097231 10.1016/j.vetpar.2006.10.001

[CR15] Feng Y, Xiao L (2011) Zoonotic potential and molecular epidemiology of *Giardia* species and giardiasis. Clin Microbiol Rev 24:110–140. 10.1128/cmr.00033-10 21233509 10.1128/CMR.00033-10 PMC3021202

[CR16] Figueiredo AM, Köster PC, Dashti A et al (2023) Molecular detection and distribution of *Giardia duodenalis* and *Cryptosporidium* spp. infections in wild and domestic animals in Portugal. Transbound Emerg Dis 2023:5849842. 10.1155/2023/5849842 40303765 10.1155/2023/5849842 PMC12017001

[CR17] Fox NJ, Marion G, Davidson RS et al (2015) Climate-driven tipping-points could lead to sudden, high-intensity parasite outbreaks. R Soc Open Sci 2:140296. 10.1098/rsos.140296 26064647 10.1098/rsos.140296 PMC4453250

[CR18] Gao H, Liang G, Su N et al (2023) Prevalence and molecular characterization of *Cryptosporidium* spp., *Giardia duodenalis*, and *Enterocytozoon bieneusi* in diarrheic and non-diarrheic calves from Ningxia, Northwestern China. Animals 13:1983. 10.3390/ani13121983 37370492 10.3390/ani13121983 PMC10295355

[CR19] Hamilton KA, Waso M, Reyneke B et al (2018) *Cryptosporidium* and *Giardia* in wastewater and surface water environments. J Environ Qual 47:1006–1023. 10.2134/jeq2018.04.0132 30272766 10.2134/jeq2018.04.0132

[CR20] Hamnes IS, Gjerde B, Robertson L (2006) Prevalence of *Giardia* and *Cryptosporidium* in dairy calves in three areas of Norway. Vet Parasitol 140:204–216. 10.1016/j.vetpar.2006.03.024 16647210 10.1016/j.vetpar.2006.03.024

[CR21] Heyworth MF (2016) *Giardia duodenalis* genetic assemblages and hosts. Parasite 23. 10.1051/parasite/2016013. 10.1051/parasite/2016013 PMC4794627 26984116

[CR22] Hoang DT, Chernomor O, von Haeseler A et al (2018) UFBoot2: improving the ultrafast bootstrap approximation. Mol Biol Evol 35:518–522. 10.1093/molbev/msx281 29077904 10.1093/molbev/msx281 PMC5850222

[CR23] Jang D-H, Cho H-C, Park Y-J et al (2023) First report of *Cryptosporidium andersoni*and risk factors associated with the occurrence of *Cryptosporidium*spp. in pre-weaned native Korean calves with diarrhea. Front Vet Sci. 10.3389/fvets.2023.1145096 37026096 10.3389/fvets.2023.1145096 PMC10070877

[CR24] Kalyaanamoorthy S, Minh BQ, Wong TKF et al (2017) Modelfinder: fast model selection for accurate phylogenetic estimates. Nat Methods 14:587–589. 10.1038/nmeth.4285 28481363 10.1038/nmeth.4285 PMC5453245

[CR25] Katoh K, Kuma K, Toh H, Miyata T (2005) MAFFT version 5: improvement in accuracy of multiple sequence alignment. Nucleic Acids Res 33:511–518. 10.1093/nar/gki198 15661851 10.1093/nar/gki198 PMC548345

[CR26] Katoh K, Rozewicki J, Yamada KD (2019) MAFFT online service: multiple sequence alignment, interactive sequence choice and visualization. Brief Bioinform 20:1160–1166. 10.1093/bib/bbx108 28968734 10.1093/bib/bbx108 PMC6781576

[CR27] Koehler AV, Korhonen PK, Hall RS et al (2017) Use of a bioinformatic-assisted primer design strategy to establish a new nested PCR-based method for *Cryptosporidium*. Parasit Vectors 10:509. 10.1186/s13071-017-2462-4 29061171 10.1186/s13071-017-2462-4 PMC5654123

[CR28] Kumar S, Stecher G, Suleski M et al (2024) MEGA12: molecular evolutionary genetic analysis version 12 for adaptive and green computing. Mol Biol Evol 41:msae263. 10.1093/molbev/msae263 39708372 10.1093/molbev/msae263 PMC11683415

[CR29] Lalle M, Pozio E, Capelli G et al (2005) Genetic heterogeneity at the *β-giardin* locus among human and animal isolates of *Giardia duodenalis* and identification of potentially zoonotic subgenotypes. Int J Parasitol 35:207–213. 10.1016/j.ijpara.2004.10.022 15710441 10.1016/j.ijpara.2004.10.022

[CR30] Louro M, Bexiga R, Da Fonseca IP, Gomes J (2024) Detection and molecular characterization of *Cryptosporidium* spp. in dairy calves in Lisbon and Tagus Valley, Portugal. Vet Parasitol Reg Stud Rep 47:100964. 10.1016/j.vprsr.2023.100964 10.1016/j.vprsr.2023.100964 38199683

[CR31] Louro M, Hernandez L, Antunes J et al (2025a) *Cryptosporidium* spp. in reptiles: detection challenges, molecular characterization and zoonotic risk. Food Waterborne Parasitol e00272. 10.1016/j.fawpar.2025.e00272 40546388 10.1016/j.fawpar.2025.e00272 PMC12182340

[CR32] Louro M, Ruano Z, Lozano J et al (2025b) Molecular characterization of *Cryptosporidium*spp. and *Giardia duodenalis*infections and impact on growth performance of Churra Galega Mirandesa lambs. Vet Parasitol 110585. 10.1016/j.vetpar.2025.110585 40882491 10.1016/j.vetpar.2025.110585

[CR33] Marbella D, Santana-Hernández KM, Rodríguez-Ponce E (2022) Small islands as potential model ecosystems for parasitology: climatic influence on parasites of feral cats. J Helminthol 96:e51. 10.1017/S0022149X22000451 35856271 10.1017/S0022149X22000451

[CR34] Massot A (2015) A agricultura do arquipélago Dos Açores (Delegação Da COMAGRI). European Parliament, Brussels

[CR35] Medeiros I, Fernandez-Novo A, Astiz S, Simões J (2021) Production and health management from grazing to confinement systems of largest dairy bovine farms in Azores: a farmers’ perspective. Animals 11:3394. 10.3390/ani11123394 34944171 10.3390/ani11123394 PMC8697991

[CR36] Nguyen L-T, Schmidt HA, von Haeseler A, Minh BQ (2015) IQ-TREE: a fast and effective stochastic algorithm for estimating maximum-likelihood phylogenies. Mol Biol Evol 32:268–274. 10.1093/molbev/msu300 25371430 10.1093/molbev/msu300 PMC4271533

[CR37] Olson ME, Goh J, Phillips M et al (1999) *Giardia* cyst and *Cryptosporidium* oocyst survival in water, soil, and cattle feces. J Environ Qual 28:1991–1996. 10.2134/jeq1999.00472425002800060040x

[CR38] Pebesma E (2018) Simple features for R: standardized support for Spatial vector data. R J 10:439–446. 10.32614/RJ-2018-009

[CR39] Pebesma E, Bivand R (2023) Spatial Data Science: With applications in R. Chapman and Hall/CRC

[CR40] Portal do INE. https://www.ine.pt/xportal/xmain?xpid=INE&xpgid=ine_indicado993res&indOcorrCod=0013527&contexto=bd&selTab=tab2.Accessed 27 July 2025

[CR41] R Core Team (2023) R: A Language and. Environment for Statistical Computing

[CR42] Read CM, Monis PT, Andrew Thompson RC (2004) Discrimination of all genotypes of *Giardia duodenalis* at the *glutamate dehydrogenase* locus using PCR-RFLP. Infect Genet Evol 4:125–130. 10.1016/j.meegid.2004.02.001 15157630 10.1016/j.meegid.2004.02.001

[CR43] Rzeżutka A, Kaupke A (2023) *Cryptosporidium* infections in asymptomatic calves up to 4 months in Poland: a cross-sectional population study. Sci Rep 13:20997. 10.1038/s41598-023-47810-5 38017032 10.1038/s41598-023-47810-5 PMC10684609

[CR44] Santin M (2020) *Cryptosporidium* and *Giardia* in ruminants. Vet Clin North Am Food Anim Pract 36:223–238. 10.1016/j.cvfa.2019.11.005 32029186 10.1016/j.cvfa.2019.11.005

[CR45] Shaw HJ, Innes EA, Morrison LJ et al (2020) Long-term production effects of clinical cryptosporidiosis in neonatal calves. Int J Parasitol 50:371–376. 10.1016/j.ijpara.2020.03.002 32277986 10.1016/j.ijpara.2020.03.002 PMC7194893

[CR46] Silva E, Almeida B, Marta-Costa AA (2019) Efficiency of the dairy farms: a study from Azores (Portugal). Eur Countrys 10:725–734. 10.2478/euco-2018-0040

[CR47] Sulaiman IM, Fayer R, Bern C et al (2003) Triosephosphate Isomerase Gene characterization and potential zoonotic transmission of *Giardia duodenalis*. Emerg Infect Dis 9:1444–1452. 10.3201/eid0911.030084 14718089 10.3201/eid0911.030084 PMC3035538

[CR48] Sulaiman IM, Hira PR, Zhou L et al (2005) Unique endemicity of cryptosporidiosis in children in Kuwait. J Clin Microbiol 43:2805–2809. 10.1128/jcm.43.6.2805-2809.2005 15956401 10.1128/JCM.43.6.2805-2809.2005 PMC1151898

[CR49] Taghipour A, Sharbatkhori M, Tohidi F et al (2022) Global prevalence of *Giardia duodenalis* in cattle: a systematic review and meta-analysis. Prev Vet Med 203:105632. 10.1016/j.prevetmed.2022.105632 35427916 10.1016/j.prevetmed.2022.105632

[CR50] Thomson S, Hamilton CA, Hope JC et al (2017) Bovine cryptosporidiosis: impact, host-parasite interaction and control strategies. Vet Res 48:42. 10.1186/s13567-017-0447-0 28800747 10.1186/s13567-017-0447-0 PMC5553596

[CR51] Trifinopoulos J, Nguyen L-T, von Haeseler A, Minh BQ (2016) W-IQ-TREE: a fast online phylogenetic tool for maximum likelihood analysis. Nucleic Acids Res 44:W232–W235. 10.1093/nar/gkw256 27084950 10.1093/nar/gkw256 PMC4987875

[CR52] Urie NJ, Lombard JE, Shivley CB et al (2018) Preweaned heifer management on US dairy operations: part III. Factors associated with *Cryptosporidium* and *Giardia* in preweaned dairy heifer calves. J Dairy Sci 101:9199–9213. 10.3168/jds.2017-14060 29859689 10.3168/jds.2017-14060

[CR53] Wickham H (2016) ggplot2: Elegant Graphics for Data Analysis. Springer-Verlag, New York

[CR54] Xiao L (2010) Molecular epidemiology of cryptosporidiosis: an update. Exp Parasitol 124:80–89. 10.1016/j.exppara.2009.03.018 19358845 10.1016/j.exppara.2009.03.018

[CR55] Xiao L, Alderisio K, Limor J et al (2000) Identification of species and sources of *Cryptosporidium* oocysts in storm waters with a small-subunit rRNA-based diagnostic and genotyping tool. Appl Environ Microbiol 66:5492–5498. 10.1128/AEM.66.12.5492-5498.2000 11097935 10.1128/aem.66.12.5492-5498.2000 PMC92489

[CR56] Xiao L, Morgan UM, Limor J et al (1999) Genetic diversity within *Cryptosporidium parvum* and related *Cryptosporidium* species. Appl Environ Microbiol 65:3386–3391. 10.1128/AEM.65.8.3386-3391.1999 10427023 10.1128/aem.65.8.3386-3391.1999 PMC91508

