# Supplementary material for: Integrated epidemiological and molecular analysis of Cryptosporidium spp. and Giardia duodenalis isolates in dairy calves from Terceira Island, Azores
Source: Parasitol Res. 2025 Dec 5;124(12):151. doi: 10.1007/s00436-025-08613-x (PMC12680760; doi:10.1007/s00436-025-08613-x)
Supplement: Supplementary file 1 — Supplementary Material 1 (DOCX 46.6 KB) [file 436_2025_8613_MOESM1_ESM.docx]

**Supplementary table 1:** Primers and PCR conditions used for the molecular detection and characterization of *Cryptosporidium* spp. and *Giardia duodenalis*

| Specie & locus | Primer 5’-3’ | Fragment size (bp) | Termocycler | | | | Source |
| --- | --- | --- | --- | --- | --- | --- | --- |
|  |  |  | 1’ program | | 2’ program | |  |
| *Cryptosporidium* spp.  SSU rRNA | F1: TTCTAGAGCTAATACATGCG | ≈ 1350 | 1  35  1 | 95°C 5min  94°C 45s  55°C 45s  72°C 60s  72°C 5min | 1  35  1 | 95°C 5min  94°C 45s  58°C 45s  72°C 60s  72°C 5min | (Xiao et al., 1999, 2000) |
|  | R1: CCCATTTCCTTCGAAACAGGA |  |  |  |  |  |  |
|  | F2: GGAAGGGTTGTATTTATTAGATAAAG | ≈ 800 |  |  |  |  |  |
|  | R2: CTCATAAGGTGCTGAAGGAGTA |  |  |  |  |  |  |
| *Cryptosporidium* spp.  gp60 | F1: ATGAGATTGTCGCTCATTATCG | ≈ 1000 | 1  35  1 | 95°C 5min  94°C 45s  52°C 45s  72°C 60s  72°C 5min | 1  35  1 | 95°C 5min  94°C 45s  52°C 45s  72°C 60s  72°C 5min | (Alves et al., 2003) |
|  | R1: TTACAACACGAATAAGGCTGC |  |  |  |  |  |  |
|  | F2: TCCGCTGTATTCTCAGCC | ≈ 850 |  |  |  |  |  |
|  | R2: GGAAGGAACGATGTATCT |  |  |  |  |  |  |
| *Cryptosporidium* spp.  LSU | F1: CGAATAGCGTTATCTTTGCTATTT | ≈ 1000 | 1  35  1 | 95°C 5min  94°C 30s  58°C 30s  72°C 60s  72°C 5min | 1  35  1 | 95°C 5min  94°C 30s  58°C 30s  72°C 30s  72°C 5min | (Koehler et al., 2017) |
|  | R1: GTCTTCCGCGAAGATCAG |  |  |  |  |  |  |
|  | F2: TTACCATGGAATYAGTTCAGC | ≈ 400 |  |  |  |  |  |
|  | R2: AACACCATTTTCTGGCCATC |  |  |  |  |  |  |
| *Giardia duodenalis*  gdh | F1: TCAACGTYAAYCGYGGYTTCCGT | - | 1  40  1 | 95°C 5min  94°C 45s  50°C 30s  72°C 60s  72°C 5min | 1  40  1 | 95°C 5min  94°C 45s  60°C 30s  72°C 45s  72°C 5min | (Read et al., 2004) |
|  | R1: GTTRTCCTTGCACATCTCC |  |  |  |  |  |  |
|  | F2: CAGTACACCTCYGCTCTCGG | 432 |  |  |  |  |  |
|  | R2: GTTRTCCTTGCACATCTCC |  |  |  |  |  |  |
| *Giardia duodenalis*  bg | F1: AAGCCCGACGACCTCACCCGCAGTGC | 753 | 1  40  1 | 95°C 5min  94°C 45s  50°C 30s  72°C 60s  72°C 5min | 1  35  1 | 95°C 5min  94°C 45s  55°C 30s  72°C 45s  72°C 5min | (Lalle et al., 2005) |
|  | R1: GAGGCCGCCCTGGATCTTCGAGACGAC |  |  |  |  |  |  |
|  | F2: GAACGAACGAGATCGAGGTCCG | 511 |  |  |  |  |  |
|  | R2: CTCGACGAGCTTCGTGTT |  |  |  |  |  |  |
| *Giardia duodenalis*  tpi | F1: AAATIATGCCTGCTCGTCG | 605 | 1  40  1 | 95°C 5min  94°C 45s  50°C 30s  72°C 60s  72°C 5min | 1  40  1 | 95°C 5min  94°C 45s  50°C 30s  72°C 60s  72°C 5min | (Sulaiman et al., 2003) |
|  | R1: CAAACCTTITCCGCAAACC |  |  |  |  |  |  |
|  | F2: CCCTTCATCGGIGGTAACTT | 530 |  |  |  |  |  |
|  | R2: GTGGCCACCACICCCGTGCC |  |  |  |  |  |  |

**Supplementary table 2:** PCR master mix recipe for *Cryptosporidium* spp. and *Giardia duodenalis* genes.

| *Cryptosporidium* spp.: SSU rRNA, gp60 | | | | | |
| --- | --- | --- | --- | --- | --- |
| Primary PCR master mix | | | **Secondary PCR master mix** | | |
| Reagent | Per 20 μL reaction (μL) | Final concentration | Reagent | Per 25 μL reaction (μL) | Final concentration |
| Master Mix^1^ | 10 | - | Master Mix^1^ | 12.5 | - |
| Primer F1 (10 μM) | 0.5 | 250 nM | Primer F2 (10 μM) | 1.25 | 500 nM |
| Primer R1 (10 μM) | 0.5 | 250 nM | Primer R2 (10 μM) | 1.25 | 500 nM |
| Distilled water | 5 | - | Distilled water | 9 | - |
| BSA^2^ (10 mg/mL) | 1 | 500 ng/μL | - | - | - |
| *Cryptosporidium* spp.: LSU rRNA; *Giardia duodenalis*: gdh, bg, tpi | | | | | |
| Primary PCR master mix | | | **Secondary PCR master mix** | | |
| Reagent | Per 20 μL reaction (μL) | Final concentration | Reagent | Per 25 μL reaction (μL) | Final concentration |
| Master Mix^1^ | 10 | - | Master Mix^1^ | 12.5 | - |
| Primer F1 (10 μM) | 1 | 500 nM | Primer F2 (10 μM) | 1.25 | 500 nM |
| Primer R1 (10 μM) | 1 | 500 nM | Primer R2 (10 μM) | 1.25 | 500 nM |
| Distilled water | 4.5 | - | Distilled water | 9 | - |
| BSA^2^ (10 mg/mL) | 0.5 | 250 ng/μL | - | - | - |

^1^Supreme NZYTaq II 2× Green Master Mix (NZYTech, Portugal); ^2^BSA= bovine serum albumin

**Supplementary table 3:** Parasite prevalence in calves by parish, with associated farm IDs and hygiene scores (Terceira Island).

| Parish | Farm ID | Higine Score | Total samples | Prevalence % (n) | | |
| --- | --- | --- | --- | --- | --- | --- |
|  |  |  |  | *Cryptosporidium* spp. | *Giardia* spp. | *Cryptosporidium* spp. and/or *Giardia* spp. |
| Agualva | 16 | 6 | 5 | 60% (n=3) | 80% (n=4) | 80% (n=4) |
| Altares | 13  4 | 4  7 | 5  5 | 40% (n=2)  20% (n=1) | 80% (n=4)  20% (n=1) | 100% (n=5)  20% (n=1) |
| Santa Luzia | 28 | 5 | 5 | 40% (n=2) | 80% (n=4) | 80% (n=4) |
| Cabo da Praia | 20 | 3 | 5 | 20% (n=1) | 20% (n=1) | 40% (n=2) |
| Cinco Ribeiras | 11 | 3 | 5 | 40% (n=2) | 20% (n=1) | 40% (n=2) |
| Doze Ribeiras | 5 | 5 | 5 | 60% (n=3) | 20% (n=1) | 60% (n=3) |
| Feteira | 8 | 7 | 5 | 60% (n=3) | 60% (n=3) | 80% (n=4) |
| Fonte do Bastardo | 23 | 7 | 5 | 80% (n=4) | 80% (n=4) | 80% (n=4) |
| Lajes | 18 | 4 | 5 | 40% (n=2) | 60% (n=3) | 100% (n=5) |
| Porto Judeu | 7 | 8 | 5 | 0 | 60% (n=3) | 60% (n=3) |
| Porto Martins | 21 | 7 | 5 | 20% (n=1) | 100% (n=5) | 100% (n=5) |
| Posto Santo | 27 | 4 | 5 | 60% (n=3) | 20% (n=1) | 80% (n=4) |
| Santa Cruz | 22 | 7 | 5 | 60% (n=3) | 60% (n=3) | 100% (n=5) |
| Quatro Ribeiras | 12 | 2 | 5 | 80% (n=4) | 80% (n=4) | 100% (n=5) |
| Raminho | 1 | 4 | 5 | 60% (n=3) | 20% (n=1) | 80% (n=4) |
| Ribeirinha | 15 | 7 | 5 | 80% (n=4) | 40% (n=2) | 80% (n=4) |
| Santa Bárbara | 25 | 5 | 5 | 40% (n=2) | 0 | 40% (n=2) |
| Serreta | 24 | 4 | 5 | 40% (n=2) | 60% (n=3) | 60% (n=3) |
| São Bartolomeu de Regatos | 19 | 7 | 5 | 0 | 80% (n=4) | 80% (n=4) |
| São Bento | 14 | 7 | 5 | 60% (n=3) | 20% (n=1) | 60% (n=3) |
| São Brás | 17 | 6 | 5 | 40% (n=2) | 20% (n=1) | 40% (n=2) |
| São Mateus da Calheta | 26 | 6 | 5 | 60% (n=3) | 20% (n=1) | 60% (n=3) |
| Terra Chã | 6 | 4 | 5 | 0 | 40% (n=2) | 40% (n=2) |
| Vila Nova | 2  3 | 2  7 | 6  6 | 50% (n=3)  33.3% (n=2) | 50% (n=3)  33.3% (n=2) | 66.7% (n=4)  50% (n=3) |
| Vila de São Sebastião | 10  9 | 1  5 | 5  5 | 40% (n=2)  0 | 0  20% (n=1) | 40% (n=2)  20% (n=1) |
